# Association of chronic kidney disease with acute clinical outcomes and hospitalization costs of cancer resection

**DOI:** 10.1371/journal.pone.0317085

**Published:** 2025-01-24

**Authors:** Sara Sakowitz, Syed Shahyan Bakhtiyar, Saad Mallick, Amulya Vadlakonda, Ifigenia Oxyzolou, Konmal Ali, Nikhil Chervu, Peyman Benharash

**Affiliations:** 1 Cardiovascular Outcomes Research Laboratories (CORELAB), University of California, Los Angeles, Los Angeles, CA, United States of America; 2 Department of Surgery, University of Colorado, Aurora, CO, United States of America; 3 Department of Surgery, University of California, Los Angeles, Los Angeles, CA, United States of America; Istanbul University-Cerrahpasa, Cerrahpasa Medical Faculty, TÜRKIYE

## Abstract

**Purpose:**

Patients with chronic kidney disease (CKD) and end-stage renal disease (ESRD) have been noted to face increased cancer incidence. Yet, the impact of concomitant renal dysfunction on acute outcomes following elective surgery for cancer remains to be elucidated.

**Methods:**

All adult hospitalizations entailing elective resection for lung, esophageal, gastric, pancreatic, hepatic, or colon cancer were identified in the 2016–2020 National Inpatient Sample. Based on stage of renal dysfunction, CKD patients were sub-classified as CKD1-3, CKD4-5, or ESRD (others: Non-CKD). Multivariable regression models were developed to assess the association of comorbid CKD/ESRD with in-hospital mortality, perioperative complications, and resource utilization.

**Results:**

Of ~515,145 patients, 32,195 (6.2%) had CKD (5.1% CKD1-3, 0.7% CKD4-5, 0.5% ESRD). The incidence of CKD among patients undergoing cancer resection increased from 5.3% in 2016 to 7.3% in 2020 (P<0.001). Following risk adjustment, CKD1-3 and CKD4-5 remained linked with similar likelihood of mortality and hospitalization costs, but greater need for blood transfusion (CKD1-3 AOR 1.21, CI 1.09–1.35; CKD4-5 AOR 1.73 CI 1.38–2.18). CKD4-5 was also associated with greater odds of infection (AOR 1.88, CI 1.34–2.62) and respiratory sequelae (AOR 1.36, CI 1.05–1.77). Further, ESRD was linked with greater odds of in-hospital mortality (AOR 2.74, CI 1.69–4.45), infection (AOR 2.31, CI 1.62–3.30) and respiratory complications (AOR 1.72, CI 1.31–2.26), as well as greater resource utilization, relative to Non-CKD.

**Conclusion:**

Comorbid renal dysfunction was linked with inferior clinical and financial outcomes following elective cancer resection. Future work is needed to develop optimal management strategies and recovery pathways for this complex cohort.

## Introduction

Chronic kidney disease (CKD) is a growing public health concern, with global incidence increasing by 89% since 1990 [1]. Despite improvements in medical management [2], CKD affects >14% of the U.S. population and has been linked with greater morbidity and reduced quality of life [3, 4]. Notably, considerable evidence has reported elevated risk of certain malignancies among CKD patients [5–11]. In a study of ~400,000 patients with renal dysfunction, Lees et al. found a ~15% increased incidence of cancer among those with mild kidney disease and a ~19% increased risk in patients moderate or severe dysfunction [12]. Prior work has suggested this observation to be attributable to underlying disease, long-term immunosuppressive therapies, lifestyle factors, closer monitoring, or advancements in dialysis that permit greater life expectancy [13, 14]. Altogether, cancer has become the second leading cause of death in this population [12].

Definitive surgical resection remains the standard of care for select patients with localized neoplasms. While some have reported CKD patients to face increased risk of mortality and complications following surgical procedures [15–17], prior literature evaluating outcomes of cancer resection among CKD patients has yielded conflicting results. Several single-institution series have suggested similar postoperative outcomes following colectomy, gastrectomy, hepatectomy, or lobectomy [18–20]. However, others have noted increased hazards of mortality in both the short- and long-term [9, 21, 22]. In addition to evaluating heterogeneous populations and limited cohorts, available literature has relied on decades-old datasets and may not be generalizable to the contemporary oncologic landscape. Accurate characterization of perioperative outcomes for cancer surgery may better inform risk stratification, patient counseling, and shared decision-making among those with CKD.

The present study examined the association of comorbid CKD with clinical and financial outcomes of major surgical resection for several malignancies. We hypothesized increasing degree of renal dysfunction to be associated with a stepwise increase in the risk of death and complications as well as costs.

## Methods

### Data source and study cohort

All elective adult (≥18 years) hospitalization records entailing for resection of colon, hepatic, gastric, esophageal, lung, or pancreatic cancer were tabulated from the 2016–2020 National Inpatient Sample (NIS) using relevant *International Classification of Diseases*, *Tenth Revision* (ICD) diagnosis and procedure codes [23]. As the largest all-payer database in the US, the NIS samples from hospitals participating in the Healthcare Cost and Utilization Project (HCUP) and provides accurate estimates for approximately 97% of all hospitalizations, each year [24].

**Fig 1:**
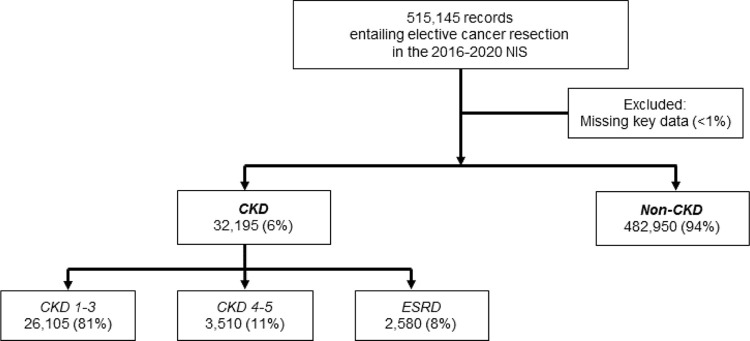
CONSORT diagram of survey-weighted estimates. Of an estimated 515,145 hospitalizations for elective cancer resection identified in the 2016–2020 NIS, 32,195 (6%) presented with comorbid CKD (others: Non-CKD). Among patients with CKD, 81% had Stage 1–3 disease, 11% Stage 4–5 disease, and 8% End-Stage Renal Disease. All estimates represent survey-weighted methodology. *NIS, National Inpatient Sample; CKD, Chronic Kidney Disease; ESRD, End-Stage Renal Disease.

Records were included for analysis if they contained both a diagnosis code for cancer and a concordant procedure code for resection. Using previously-validated *ICD* codes and in line with criteria established by the National Kidney Foundation criteria, patients were subsequently grouped by stage of renal dysfunction as CKD1-3, CKD4-5, or ESRD (others: Non-CKD) [17, 25]. Those missing key data regarding age, sex, or mortality were not evaluated (Fig 1).

### Variable definitions and study outcomes

The HCUP Data Dictionary was used to define relevant patient and hospital characteristics [26]. Patient burden of chronic disease was numerically depicted via the van Walraven modification of the Elixhauser Comorbidity Index. Comorbidities and perioperative complications were identified using appropriate *ICD* codes, as reported in prior work [27]. Complications were classified as cardiac (cardiac arrest, myocardial infarction, tamponade, ventricular fibrillation, ventricular tachycardia, tamponade), cerebrovascular (acute ischemic events, intracranial hemorrhage, stroke), infectious (sepsis or surgical site infection), intraoperative (accidental puncture or hemorrhage), and respiratory (acute respiratory distress syndrome, pneumonia, pneumothorax, prolonged ventilation, respiratory failure), or acute kidney injury (AKI). Operative approach was classified as open or minimally-invasive.

Hospitalization costs were ascertained by application of center-specific cost-to-charge ratios to overall charges followed by inflation adjustment using the 2020 Personal Healthcare Price Index [28].

The primary outcome of this study was in-hospital mortality following cancer resection. We secondarily considered incidence of perioperative complications, duration of hospitalization (LOS), costs, and discharge to non-home facilities.

### Statistical analysis

Normally-distributed continuous variables are reported as means with standard deviation (SD), while non-normally distributed factors are detailed as medians with interquartile range (IQR). Categorical variables are presented as group proportions (%). The significance of intergroup comparisons was assessed via the Mann-Whitney *U*, adjusted Wald, and Pearson χ^2^ tests, as appropriate. Similarly, the significance of temporal trends was considered using Cuzick’s nonparametric test (nptrend) [29].

Multivariable models were fit to consider the independent association of comorbid CKD with clinical and financial outcomes of interest. Covariates were chosen for inclusion using elastic net regularization, which minimizes factor collinearity and model overfitting via a penalized least-squares approach [30]. Factors included in regression models were patient age, sex, Elixhauser comorbidity index, income, race, insurance coverage, cancer resection type, frailty, hospital teaching status, and the presence of comorbid congestive heart failure, cardiac arrhythmias, coagulopathic disorders, cerebrovascular disease, pulmonary circulation disorders, or peripheral vascular disease.

Goodness of fit was assessed using model receiver operating characteristics or the coefficient of determination, as appropriate. Outcomes are reported as adjusted odds ratio (AOR) or β-coefficient (β) for logistic and linear regression models, respectively.

As a sensitivity analysis, we applied entropy balancing to address potential differences in innate cohort characteristics and generate two balanced groups for analysis. In short, entropy balancing utilizes pseudo-propensity scores to search for optimal sample weights to balance covariates, while retaining all patients for analysis [31]. This method has been demonstrated to offer a more robust, and less biased, approach to covariate balance than propensity matching [32].

All statistical analyses were performed using Stata 16.1 (StataCorp, LLC, College Station, TX). Statistical significance was set at α = 0.05. As the NIS is fully deidentified, this study was deemed exempt from full review by the Institutional Review Board at the University of California, Los Angeles.

## Results

### Patient characteristics

**Fig 2:**
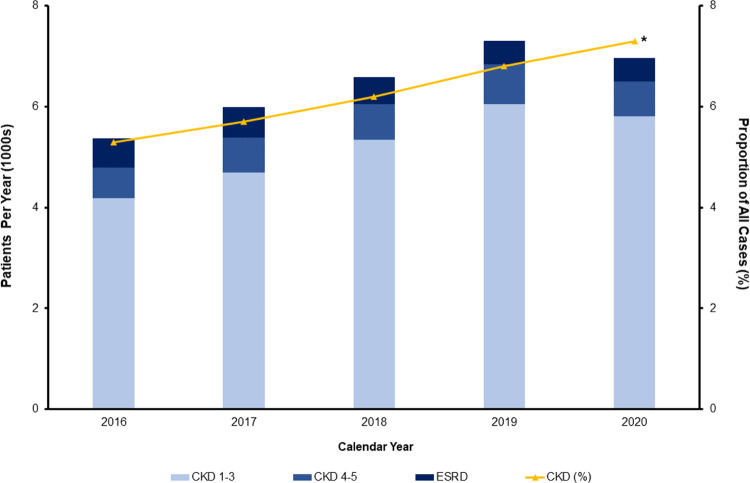
Trends of comorbid chronic kidney disease among patients undergoing elective cancer resection. The overall proportion of patients presenting with comorbid CKD increased over the study period, from 5.3% in 2016 to 7.3% in 2020 (P for trend<0.001, yellow line). Specifically, the proportion of patients undergoing cancer resection with concurrent CKD1-3 increased from 4.1 to 6.1% (P for trend<0.001), while the proportion with CKD4-5 increased from 0.6% to 0.7% (P for trend = 0.05). There was no significant change in the proportion of patients presenting with ESRD from 2016 to 2020 (0.5 to 0.5%, P for trend = 0.13). * indicates statistical significance, p<0.01.

Of an estimated 515,145 hospitalizations identified within the 2016–2020 NIS, 32,195 (6.2%) had concomitant CKD. Among the CKD cohort, 26,105 (81.1%) had CKD1-3 disease, 3,510 (10.9%) CKD4-5 disease, and 2,580 (8.0%) ESRD. The proportion of patients presenting for elective cancer resection with concurrent CKD increased significantly from 5.3% in 2016 to 7.3% in 2020 (P for trend<0.001). Specifically, the proportion with CKD1-3 and CKD4-5, but not ESRD, increased from 2016 to 2020 (Fig 2).

Compared to Non-CKD, CKD patients were, on average, older (CKD1-3 75 [69–81] vs CKD4-5 74 [67–81] vs ESRD 68 [62–74] vs Non-CKD 67 years [59–74], P<0.001), less frequently female (41.0 vs 42.5 vs 34.1 vs 49.1%, P<0.001), and more commonly of Black race (11.5 vs 15.6 vs 30.6 vs 9.3%, P<0.001). CKD patients had a higher median Elixhauser comorbidity index, compared to Non-CKD (5 [4–6] vs 6 [5–7] vs 5 [5–7] vs 3 [2–4], P<0.001). Additionally, the CKD group more frequently presented with comorbid coronary artery disease (28.5 vs 29.3 vs 23.1 vs 14.4%, P<0.001), diabetes (42.3 vs 47.3 vs 50.6 vs 21.8%, P<0.001), and peripheral vascular disease (11.1 vs 9.8 vs 8.1 vs 4.9%, P<0.001). Further, the CKD cohort was more often treated at metropolitan non-teaching instructions, relative to Non-CKD (15.2 vs 15.7 vs 15.5 vs 14.1%, P = 0.005).

Patients with CKD more frequently underwent colectomy for colon cancer (56.4 vs 62.7 vs 58.1 vs 51.9%, P<0.001) and hepatectomy for hepatocellular carcinoma (3.1 vs 3.3 vs 5.0 vs 2.9%, P<0.001). A complete characterization of the study cohort is reported in Table 1.

**Table 1:** Demographic, clinical, and hospital characteristics stratified by degree of CKD.

	*Non-CKD* (n = 482,950)	*CKD 1–3* (n = 26,105)	*CKD 4–5* (n = 3,510)	*ESRD* (n = 2,580)	*P-value*
Age (years [IQR])	67 [59–74]	75 [69–81]	74 [67–81]	68 [62–74]	
Female (%)	49.1	41.0	42.5	34.1	<0.001
Elixhauser Comorbidity Index (median [IQR])	3 [2–4]	5 [4–6]	6 [5–7]	5 [5–7]	
Frail (%)	7.9	11.9	13.0	15.5	<0.001
*Resection Type (%)*					<0.001
Colectomy for Colon Cancer	51.9	56.4	62.7	58.1	
Esophagectomy for Esophageal Cancer	1.4	0.7	0.4	0.8	
Gastrectomy for Gastric Cancer	7.2	6.1	5.4	9.1	
Hepatectomy for Hepatocellular Cancer	2.9	3.1	3.3	5.0	
Lobectomy for Lung Cancer	26.3	25.2	23.6	19.6	
Pancreatectomy for Pancreatic Cancer	10.3	8.4	4.6	7.4	
*Race (%)*					<0.001
White	76.6	77.7	74.4	51.6	
Black	9.3	11.5	15.6	30.6	
Hispanic	7.0	5.5	5.2	10.5	
Asian/Pacific Islander	4.0	3.0	2.4	4.5	
Other	3.1	2.3	2.5	2.8	
*Income percentile (%)*					<0.001
>75%	24.5	22.2	22.0	17.4	
51–75%	25.4	27.0	21.6	24.3	
26–50%	26.2	27.2	27.8	25.2	
0–25%	23.9	23.6	28.5	33.1	
*Insurance coverage (%)*					<0.001
Private	34.5	13.9	13.8	13.4	
Medicare	55.2	81.4	80.6	82.2	
Medicaid	6.7	2.6	4.6	2.7	
Other Payer	3.5	2.0	1.0	1.7	
*Comorbidities (%)*					
Coronary artery disease	14.4	28.5	29.3	23.1	<0.001
Congestive heart failure	5.4	20.8	31.8	28.7	<0.001
Diabetes	21.8	42.3	47.3	50.6	<0.001
Peripheral vascular disease	4.9	11.1	9.8	8.1	<0.001
Pulmonary circulation disorders	1.7	3.7	5.7	6.0	<0.001
Cardiac arrhythmias	18.2	30.1	29.2	22.5	<0.001
Anemia	6.2	11.6	11.3	5.6	<0.001
Coagulopathy	3.5	5.5	5.7	7.9	<0.001
Cerebrovascular disorders	3.1	4.9	7.7	9.1	<0.001
*Hospital teaching status (%)*					0.005
Non-Metropolitan	5.5	6.5	5.8	4.1	
Metropolitan Non-Teaching	14.1	15.2	15.7	15.5	
Metropolitan Teaching	80.4	78.2	78.5	80.4	

Reported as proportions unless otherwise noted. Statistical significance was set at α = 0.05.

*IQR, interquartile range; *CKD*, Chronic Kidney Disease; *ESRD*, End-Stage Renal Disease

### Unadjusted outcomes

Bivariate analysis is detailed in S1 Table. Stepwise increases in mortality, perioperative complications, and non-home discharge were noted with advancing degree of CKD. Similarly, median hospitalization costs increased from $21,600 (Non-CKD) to $23,900 (CKD1-3) to $25,200 (CKD4-5) to $26,900 (ESRD).

### Risk-adjusted outcomes

**Fig 3:**
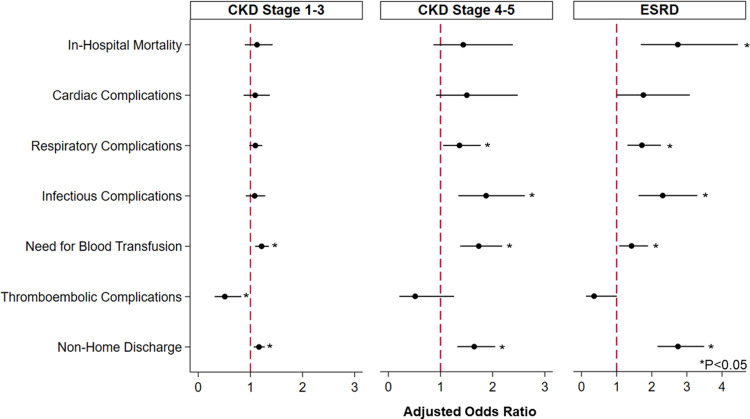
Association of CKD with perioperative outcomes. Following risk adjustment and with Non-CKD as reference, comorbid CKD was linked with greater likelihood of certain perioperative complications, need for blood transfusion, and non-home discharge, following elective cancer resection. * indicates statistical significance, P<0.05. Error bars represent 95% confidence intervals. *CKD, Chronic Kidney Disease.

Following risk adjustment, comorbid CKD remained independently associated with several perioperative outcomes of interest (Fig 3).

CKD1-3 was linked with similar in-hospital mortality (AOR 1.13, CI 0.89–1.43) and reduced odds of thrombotic complications (AOR 0.51, CI 0.31–0.82), but greater need for blood transfusion (AOR 1.21, CI 1.09–1.35). Additionally, these patients faced similar LOS and costs but higher odds of non-home discharge (AOR 1.16, CI 1.06–1.28). CKD4-5 was associated with greater odds of infection (AOR 1.88, CI 1.34–2.62), respiratory sequelae (AOR 1.36, CI 1.05–1.77), and need for blood transfusion (AOR 1.73 CI 1.38–2.18). Further, these patients experienced incremental increases in LOS (β+1.34 days, CI +0.73–1.95) and hospitalization expenditures (β+ $5,200, CI +$3,000–7,410), and greater likelihood of non-home discharge (AOR 1.65, CI 1.32–2.05). Lastly, ESRD remained associated with increased odds of in-hospital mortality (AOR 2.74, CI 1.69–4.45), as well as infectious (AOR 2.31, CI 1.62–3.30) and respiratory (AOR 1.72, CI 1.31–2.26) complications and need for blood transfusion (AOR 1.42, CI 1.07–1.90). These patients demonstrated significant increases in adjusted LOS (β+ 5.20 days; CI +3.00–7.41), costs (β+ $25,330; CI +$14,210–36,440), and likelihood of non-home discharge (AOR 2.75, CI 2.16–3.49) (Table 2).

**Table 2:** Adjusted patient outcomes stratified by degree of CKD.

	*CKD 1–3*	*P-value*	*CKD 4–5*	*P-value*	*ESRD*	*P-value*
**Clinical outcomes**						
In-hospital mortality	1.13 [0.89–1.43]	0.32	1.44 [0.86–2.39]	0.16	2.74 [1.69–4.45]	<0.001
Acute kidney injury	3.18 [2.92–3.46]	<0.001	6.49 [5.42–7.77]	<0.001	-	-
Cardiac complications	1.09 [0.87–1.37]	0.46	1.50 [0.91–2.48]	0.11	1.76 [1.01–3.09]	0.05
Infectious complications	1.08 [0.91–1.28]	0.37	1.88 [1.34–2.62]	<0.001	2.31 [1.62–3.30]	<0.001
Respiratory complications	1.10 [0.98–1.23]	0.12	1.36 [1.05–1.77]	0.02	1.72 [1.31–2.26]	<0.001
Blood transfusion	1.21 [1.09–1.35]	<0.001	1.73 [1.38–2.18]	<0.001	1.42 [1.07–1.90]	0.02
Thrombotic complication	0.51 [0.31–0.82]	0.006	0.51 [0.21–1.26]	0.15	0.36 [0.13–1.01]	0.05
Stroke complications	1.42 [0.83–2.41]	0.20	1.40 [0.44–4.49]	0.57	1.35 [0.33–5.51]	0.67
Non-home discharge	1.16 [1.06–1.28]	0.001	1.65 [1.32–2.05]	<0.001	2.75 [2.16–3.49]	<0.001
**Resource utilization**						
Length of stay (days) [IQR]	+0.00 [-0.19, 0.19]	1.00	+1.34 [0.73, 1.95]	<0.001	+5.20 [3.00, 7.41]	<0.001
Cost (USD $1,000) [IQR]	+0.52 [-0.27, 1.32]	0.20	+3.60 [0.91, 6.28]	0.009	+25.33 [+14.21, 36.44]	<0.001

Outcomes reported as Adjusted Odds Ratio (AOR) with 95% confidence intervals (95% CI). ESRD omitted from regression models for acute kidney injury due to collinearity.

*IQR, interquartile range; *USD*, United States dollar; *CKD*, Chronic Kidney Disease; *ESRD*, End-Stage Renal Disease

### Outcomes stratified by resection type

**Fig 4:**
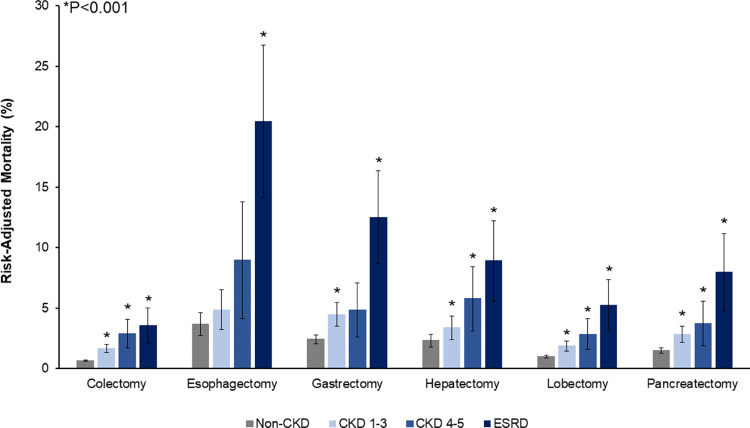
Risk-adjusted mortality of CKD cohorts stratified by resection type. A stepwise increase was noted in the adjusted risk of in-hospital mortality with greater degree of kidney disease. * indicates significance, P<0.001. *CKD, Chronic Kidney Disease; ESRD, End-Stage Renal Disease.

Subsequently we considered the association of CKD with risk-adjusted rates of in-hospital mortality and costs, stratified by cancer resection type. Relative to Non-CKD, increasing renal dysfunction was linked with greater in-hospital mortality following colectomy (CKD1-3 1.7 vs CKD4-5 2.9 vs ESRD 3.6 vs Non-CKD 0.7%, P<0.001), hepatectomy (3.4 vs 5.8 vs 8.9 vs 2.3%, P<0.001), lobectomy (1.9 vs 2.9 vs 5.2 vs 1.0%, P<0.001), and pancreatectomy (2.8 vs 3.7 vs 8.0 vs 1.5%, P<0.001). ESRD was also associated with greater mortality following esophagectomy (ESRD 20.4 vs Non-CKD 3.7%, P<0.001) and gastrectomy (12.5 vs 2.4%, P<0.001) (Fig 4).

**Fig 5:**
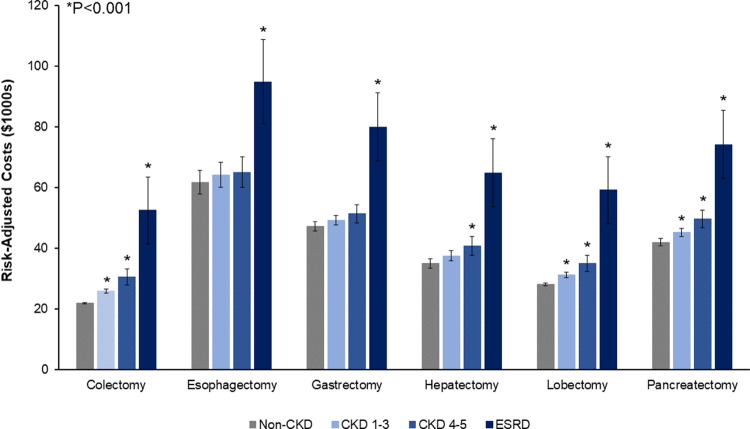
Risk-adjusted costs of CKD cohorts stratified by resection type. Increasing degree of renal dysfunction was associated with stepwise increases in hospitalization expenditures following colectomy, lobectomy, and pancreatectomy. Comorbid ESRD remained linked with greater costs across operations. * indicates statistical significance, P<0.001. *CKD, Chronic Kidney Disease; ESRD, End-Stage Renal Disease.

Evaluating costs, stepwise increases in renal disease were linked with greater costs for colectomy (CKD1-3 $25,947 vs CKD4-5 30,620 vs ESRD 52,496 vs Non-CKD 21,938, P<0.001), lobectomy ($31,273 vs 35,078 vs 59,193 vs 28,177, P<0.001), and pancreatectomy ($45,203 vs 49,595 vs 74,176 vs 42,007, P<0.001). Relative to Non-CKD, ESRD was additionally associated with greater costs for esophagectomy (ESRD $94,787 vs Non-CKD 61,751, P<0.001), gastrectomy ($79,921 vs 47,223, P<0.001), and hepatectomy ($64,812 vs 34,953, P<0.001) (Fig 5).

### Sensitivity analysis

Following entropy balancing, comorbid CKD or ESRD remained significantly associated with greater odds of in-hospital mortality (AOR 1.44, CI 1.18–1.76), as well as perioperative infection (AOR 1.48, CI 1.28–1.71), respiratory complications (AOR 1.25, CI 1.13–1.37), and required blood transfusion (AOR 1.27, CI 1.14–1.42). No difference in likelihood of cardiac complications (AOR 1.15, CI 0.93–1.43) or thromboembolism (AOR 0.71, CI 0.50–1.01) was noted. In addition to a 28% increase in relative risk of non-home discharge (AOR 1.28, CI 1.18–1.39), concurrent CKD/ESRD was linked with greater LOS (β +0.71 days, CI +0.45–0.98) and hospitalization costs (β +$3,800, CI +$2,600–5,000).

## Discussion

In the present work, we characterized the association of comorbid renal disease with select acute clinical and financial outcomes following elective resection for cancer. After adjusting for relevant patient and hospital factors, increasing CKD burden remained linked with greater odds of perioperative complications and need for blood transfusion. Additionally, advanced renal dysfunction and ESD were associated with greater resource utilization and likelihood of non-home discharge. Given the rising national prevalence of CKD, several of these findings merit further discussion.

Patients with chronic renal disease represent a growing, and complex, oncologic surgical cohort. CKD is well recognized to have multi-organ repercussions, with disruptions in intracellular fluid balance and alterations in acid/base dynamics engendering full-body physiologic consequences. Assumed to face increased postoperative morbidity risk due to broad metabolic and coagulopathic dysfunction, these patients also present with myriad conditions that may underlie or contribute to renal failure [16]. Indeed, in line with prior work [33, 34], we found patients with renal dysfunction to present with significantly greater comorbidity burden, relative to those without kidney disease. After adjusting for such comorbidities and stratifying by operation, we noted CKD patients to demonstrate a trend towards increasing mortality, with the greatest risk among ESRD patients.

In addition, we report advanced CKD and ESRD to be associated with increased odds of postoperative infection and pulmonary sequelae. These findings build on prior work that has associated CKD with three to four fold elevation in the incidence of infection, relative to the general population [35, 36]. This dramatic risk is thought to stem from uremia-induced depression of both the innate and adaptive immune systems, complicated by malnutrition, impaired glucose metabolism, and iron overload, as well as long-term use of powerful immunosuppressive drugs to control underlying disease [37]. While patients reliant on dialysis face greatest risk due to required vascular access [38], susceptibility to infection has been demonstrated across the range of CKD severity [39] Similarly, with reduced ability to manage overall fluid balance, these patients face baseline elevated risk for pulmonary edema, which can quickly advance to acute respiratory distress syndrome or pneumonia. Given this established vulnerability, future work is needed to optimize hospital practices and reduce the intra-hospital incidence and spread of infection. For example, the development and implementation of electronic health record-based order sets could guide decisions regarding appropriate perioperative antibiotic prophylaxis. Similarly, the establishment of postsurgical protocols regarding pulmonary hygiene and fluid management could help mitigate risk of pulmonary sequelae. In addition, given low reported vaccination rates among those with CKD, clinicians should actively encourage patients to get vaccinated against known fomites, such as pneumococcal pneumonia or influenza. Combined, these efforts could lead to reduce infection transmission and severity among these at-risk patients [40, 41].

Notably, we identified CKD patients to demonstrate significantly greater need for blood transfusions. While largely expected due to preexisting anemia and coagulopathy that may arise from platelet dysfunction [42, 43], this finding also suggests an opportunity for improvement in pre- and intraoperative management. Preoperative utilization of erythropoietin to maintain appropriate hemoglobin levels, or preemptive transfusion in combination with dialysis have both been used successfully in patients with advanced kidney disease [44]. Reducing overall transfusion volume or frequency may be especially beneficial for patients undergoing resection for cancer, given that blood transfusions have been independently linked with inferior outcomes [45]. Ultimately, future prospective research is warranted to identify optimal multimodal preoperative management, red blood cell transfusion strategies, and potentially improved hemostasis for surgical patients with CKD. These findings could subsequently be integrated into standardized in-hospital perioperative blood transfusion protocols, which could be implemented upon patient admission for surgical management. Greater vigilance regarding perioperative fluid balance may also mitigate the expected increase in relative risk of acute kidney injury faced by CKD patients [46]. As the development of acute renal injury has been independently linked with faster progression of renal dysfunction [47], interventions to mitigate AKI incidence during hospitalization could have significant implications for patients’ renal function in the longer term. Further, as our study exclusively considered acute in-hospital outcomes, future investigations could build on our findings and more broadly evaluate the association of CKD with longer-term complications and survival.

Finally, we found moderate/severe renal disease and ESRD to be linked with significantly greater resource utilization. In particular, ESRD was associated with a 5-day incremental increase in duration of hospitalization and a ~$25,000 median increase in per-patient costs. We attribute much of this cost differential to more intensive medical and surgical management, including the need for in-hospital dialysis or admission to the intensive care unit [48]. The development and implementation of in-hospital pathways to optimize care, including infection prophylaxis, more active pulmonary toilet, and improved blood transfusion protocols, may contribute to lowering this cost burden. Importantly, patients of increasing CKD severity also faced higher adjusted risk of non-home discharge. This finding suggests potential for the early, preoperative identification of individuals who are most likely to require discharge to skilled nursing or acute rehabilitation facilities. With earlier discharge planning, post-hospital arrangements could be made without unnecessarily prolonging hospitalization [49]. This may benefit patients in two distinct manners: First, in eliminating extended exposure to the risk of nosocomial infections in the hospital, and second, in reducing overall expenditures [50]. While we did not assess readmission in the present work, future studies could consider the impact of comprehensive discharge planning on outcomes, re-hospitalization, and follow-up care.

This work presents with certain limitations inherent to its retrospective design. As a national database, the NIS permits a large, representative sample for analysis, but does not capture granular physiologic or laboratory data points. Individual glomerular filtration rates and serum creatinine values were unavailable, as was duration of renal dysfunction. However, we used previously-validated administrative codes for CKD stage to stratify subgroups by degree of kidney disease. The NIS does not include information regarding hospital-specific care pathways, perioperative optimization strategies, or nurse-to-patient ratios, and so we could not consider these factors in our analysis. We could not evaluate factors before hospital admission, including the influence of insurance coverage on access to CKD or cancer care. Additionally, data beyond hospital discharge was not recorded. Building on our work, future studies are needed to consider the association of CKD with longer-term sequelae of major oncologic resections. Despite these limitations, we applied robust statistical methods among a large, nationally-representative sample to consider the association of comorbid CKD on outcomes following elective cancer operations.

## Conclusion

Comorbid CKD was linked with greater perioperative complications and non-home discharge following elective cancer resection. Further, ESRD was associated with significantly increased odds of mortality and resource utilization, relative to patients without renal dysfunction. Ultimately, our work contributes a contemporary perspective on the risk of acute morbidity following cancer resection among patients with CKD. Yet, given the growing population of patients with renal disease, novel strategies and care pathways are needed to improve perioperative outcomes among those undergoing resection for cancer.

## Supporting information

S1 Table Unadjusted patient outcomes stratified by degree of CKD. Outcomes reported as proportion (%). *IQR, interquartile range; *USD*, United States dollar; *CKD*, Chronic Kidney Disease; *ESRD*, End-Stage Renal Disease.
(DOCX)

## References

[1] Xie Y, Bowe B, Mokdad AH, et al. Analysis of the Global Burden of Disease study highlights the global, regional, and national trends of chronic kidney disease epidemiology from 1990 to 2016. Kidney Int. 2018;94(3):567–581. doi: 10.1016/j.kint.2018.04.011 30078514

[2] Kalantar-Zadeh K, Jafar TH, Nitsch D, Neuen BL, Perkovic V. Chronic kidney disease. The Lancet. 2021;398(10302):786–802. doi: 10.1016/S0140-6736(21)00519-5 34175022

[3] Perlman RL, Finkelstein FO, Liu L, et al. Quality of life in chronic kidney disease (CKD): A Cross-sectional analysis in the Renal Research Institute-CKD study. Am J Kidney Dis. 2005;45(4):658–666. doi: 10.1053/j.ajkd.2004.12.021 15806468

[4] Wong G, Howard K, Chapman J, et al. How do people with chronic kidney disease value cancer-related quality of life? Nephrology. 2012;17(1):32–41. doi: 10.1111/j.1440-1797.2011.01531.x 22017753

[5] Lowrance WT, Ordoñez J, Udaltsova N, Russo P, Go AS. CKD and the risk of incident cancer. J Am Soc Nephrol JASN. 2014;25(10):2327–2334. doi: 10.1681/ASN.2013060604 24876115 PMC4178430

[6] Wong G, Hayen A, Chapman JR, et al. Association of CKD and cancer risk in older people. J Am Soc Nephrol JASN. 2009;20(6):1341–1350. doi: 10.1681/ASN.2008090998 19406977 PMC2689896

[7] Wu MY, Chang TC, Chao TY, Huang MT, Lin HW. Risk of colorectal cancer in chronic kidney disease: A Matched cohort study based on administrative data. Ann Surg Oncol. 2013;20(12):3885–3891. doi: 10.1245/s10434-013-3065-8 23807660

[8] Park S, Lee S, Kim Y, et al. Risk of cancer in pre-dialysis chronic kidney disease: A Nationwide population-based study with a matched control group. Kidney Res Clin Pract. 2019;38(1):60–70. doi: 10.23876/j.krcp.18.0131 30866180 PMC6481964

[9] Wei YF, Chen JY, Lee HS, Wu JT, Hsu CK, Hsu YC. Association of chronic kidney disease with mortality risk in patients with lung cancer: A Nationwide Taiwan population-based cohort study. BMJ Open. 2018;8(1):e019661. doi: 10.1136/bmjopen-2017-019661 29371286 PMC5786081

[10] Chen B, Fan VY, Chou YJ, Kuo CC. Costs of care at the end of life among elderly patients with chronic kidney disease: patterns and predictors in a nationwide cohort study. BMC Nephrol. 2017;18(1):36. doi: 10.1186/s12882-017-0456-2 28122500 PMC5267416

[11] Oh HJ, Lee HA, Moon CM, Ryu DR. Incidence risk of various types of digestive cancers in patients with pre-dialytic chronic kidney disease: A Nationwide population-based cohort study. PLOS ONE. 2018;13(11):e0207756. doi: 10.1371/journal.pone.0207756 30458033 PMC6245741

[12] Lees JS, Ho F, Parra-Soto S, et al. Kidney function and cancer risk: An Analysis using creatinine and cystatin C in a cohort study. eClinicalMedicine. 2021;38:101030. doi: 10.1016/j.eclinm.2021.101030 34505030 PMC8413238

[13] Hu M, Wang Q, Liu B, et al. Chronic kidney disease and cancer: Inter-relationships and mechanisms. Front Cell Dev Biol. 2022;10. Accessed November 9, 2023. doi: 10.3389/fcell.2022.868715 35663394 PMC9158340

[14] Lees JS, Elyan BMP, Herrmann SM, Lang NN, Jones RJ, Mark PB. The ‘other’ big complication: How chronic kidney disease impacts on cancer risks and outcomes. Nephrol Dial Transplant. 2023;38(5):1071–1079. doi: 10.1093/ndt/gfac011 35090037 PMC10157781

[15] Gaber AO, Moore LW, Aloia TA, et al. Cross-sectional and case-control analyses of the association of kidney function staging with adverse postoperative outcomes in general and vascular surgery. Ann Surg. 2013;258(1):169. doi: 10.1097/SLA.0b013e318288e18e 23478526

[16] Newman LA, Mittman N, Hunt Z, Alfonso AE. Survival among chronic renal failure patients requiring major abdominal surgery. J Am Coll Surg. 1999;188(3):310–314. doi: 10.1016/s1072-7515(98)00308-1 10065821

[17] Dobaria V, Hadaya J, Richardson S, et al. Clinical and financial impact of chronic kidney disease in emergency general surgery operations. Surg Open Sci. 2022;10:19–24. doi: 10.1016/j.sopen.2022.05.013 35846391 PMC9283654

[18] Lu MS, Chen MF, Lin CC, et al. Is chronic kidney disease an adverse factor in lung cancer clinical outcome? A ppropensity score matching study. Thorac Cancer. 2017;8(2):106–113. doi: 10.1111/1759-7714.12414 28207203 PMC5334301

[19] Currie A, Malietzis G, Askari A, et al. Impact of chronic kidney disease on postoperative outcome following colorectal cancer surgery. Colorectal Dis. 2014;16(11):879–885. doi: 10.1111/codi.12665 24836209

[20] Toshima T, Shirabe K, Yoshiya S, et al. Outcome of hepatectomy for hepatocellular carcinoma in patients with renal dysfunction. HPB. 2012;14(5):317–324. doi: 10.1111/j.1477-2574.2012.00452.x 22487069 PMC3384851

[21] Nozawa H, Kitayama J, Sunami E, Watanabe T. Impact of chronic kidney disease on outcomes of surgical resection for primary colorectal cancer: A Retrospective cohort review. Dis Colon Rectum. 2012;55(9):948. doi: 10.1097/DCR.0b013e3182600db7 22874601

[22] Sakurai K, Kubo N, Tamamori Y, et al. Impact of chronic kidney disease on the short- and long-term outcomes of laparoscopic gastrectomy for gastric cancer patients. PLOS ONE. 2021;16(4):e0250997. doi: 10.1371/journal.pone.0250997 33930090 PMC8087092

[23] Pan CS, Sanaiha Y, Hadaya J, Lee C, Tran Z, Benharash P. Venous thromboembolism in cancer surgery: A report from the nationwide readmissions database. Surg Open Sci. 2022;9:58–63. doi: 10.1016/j.sopen.2022.04.005 35669894 PMC9166654

[24] Healthcare Cost and Utilization Project. Overview of the National (Nationwide) Inpatient Sample (NIS). Accessed December 15, 2021. https://www.hcup-us.ahrq.gov/nisoverview.jsp

[25] Sanaiha Y, Kavianpour B, Downey P, Morchi R, Shemin RJ, Benharash P. National study of index and readmission mortality and costs for thoracic endovascular aortic repair in patients with renal disease. Ann Thorac Surg. 2020;109(2):458–464. doi: 10.1016/j.athoracsur.2019.05.071 31336063

[26] Healthcare Cost and Utilization Project. NIS Description of Data Elements. Accessed March 15, 2022. https://www.hcup-us.ahrq.gov/db/nation/nis/nisdde.jsp

[27] Madrigal J, Tran Z, Hadaya J, Sanaiha Y, Benharash P. Impact of chronic lymphocytic leukemia on outcomes and readmissions after cardiac operations. Ann Thorac Surg. Published online 2021. doi: 10.1016/j.athoracsur.2021.07.059 34437856

[28] Agency for Healthcare Research and Quality. Using Appropriate Price Indices for Expenditure Comparisons. Accessed March 15, 2022. https://meps.ahrq.gov/about_meps/Price_Index.shtml

[29] Cuzick J. A Wilcoxon-type test for trend. Stat Med. 1985;4(1):87–90. doi: 10.1002/sim.4780040112 3992076

[30] Zou H, Hastie T. Regularization and variable selection via the elastic net. J R Stat Soc B. 2005;67(2):301–320.

[31] Hainmueller J. Entropy balancing for causal effects: A multivariate reweighting method to produce balanced samples in observational studies. Polit Anal. 2012;20(1):25–46. doi: 10.1093/pan/mpr025

[32] Zhao Q, Percival D. Entropy Balancing is Doubly Robust. J Causal Inference. 2017;5(1). doi: 10.1515/jci-2016-0010

[33] Tonelli M, Wiebe N, Culleton B, et al. Chronic kidney disease and mortality risk: A Systematic review. J Am Soc Nephrol. 2006;17(7):2034. doi: 10.1681/ASN.2005101085 16738019

[34] Goodkin DA, Bragg-Gresham JL, Koenig KG, et al. Association of comorbid conditions and mortality in hemodialysis patients in Europe, Japan, and the United States: The Dialysis Outcomes and Practice Patterns Study (DOPPS). J Am Soc Nephrol. 2003;14(12):3270. doi: 10.1097/01.asn.0000100127.54107.57 14638926

[35] Ishigami J, Grams ME, Chang AR, Carrero JJ, Coresh J, Matsushita K. CKD and risk for hospitalization with infection: The Atherosclerosis Risk in Communities (ARIC) Study. Am J Kidney Dis Off J Natl Kidney Found. 2017;69(6):752–761. doi: 10.1053/j.ajkd.2016.09.018 27884474 PMC5438909

[36] Narayanan M. The Many faces of infection in CKD: Evolving paradigms, insights, and novel therapies. Adv Chronic Kidney Dis. 2019;26(1):5–7. doi: 10.1053/j.ackd.2018.10.001 30876617

[37] Kato S, Chmielewski M, Honda H, et al. Aspects of immune dysfunction in end-stage renal disease. Clin J Am Soc Nephrol CJASN. 2008;3(5):1526–1533. doi: 10.2215/CJN.00950208 18701615 PMC4571158

[38] Ishani A, Collins AJ, Herzog CA, Foley RN. Septicemia, access and cardiovascular disease in dialysis patients: The USRDS Wave 2 Study1. Kidney Int. 2005;68(1):311–318. doi: 10.1111/j.1523-1755.2005.00414.x 15954922

[39] James MT, Laupland KB, Tonelli M, et al. Risk of bloodstream infection in patients with chronic kidney disease not treated with dialysis. Arch Intern Med. 2008;168(21):2333–2339. doi: 10.1001/archinte.168.21.2333 19029498

[40] Krueger KM, Ison MG, Ghossein C. Practical guide to vaccination in all stages of CKD, including patients treated by dialysis or kidney transplantation. Am J Kidney Dis. 2020;75(3):417–425. doi: 10.1053/j.ajkd.2019.06.014 31585683

[41] Ma BM, Yap DYH, Yip TPS, Hung IFN, Tang SCW, Chan TM. Vaccination in patients with chronic kidney disease—Review of current recommendations and recent advances. Nephrology. 2021;26(1):5–11. doi: 10.1111/nep.13741 32524684

[42] Acedillo RR, Shah M, Devereaux PJ, et al. The Risk of perioperative bleeding in patients with chronic kidney disease: A Systematic review and meta-analysis. Ann Surg. 2013;258(6):901. doi: 10.1097/SLA.0000000000000244 24169162

[43] Augustin ID, Yeoh TY, Sprung J, Berry DJ, Schroeder DR, Weingarten TN. Association between chronic kidney disease and blood transfusions for knee and hip arthroplasty surgery. J Arthroplasty. 2013;28(6):928–931. doi: 10.1016/j.arth.2013.02.004 23518427

[44] Park BJ, Shin S, Kim HK, Choi YS, Kim J, Shim YM. Surgical treatment for non-small cell lung cancer in patients on hemodialysis due to chronic kidney disease: Clinical outcome and intermediate-term results. Korean J Thorac Cardiovasc Surg. 2015;48(3):193–198. doi: 10.5090/kjtcs.2015.48.3.193 26078926 PMC4463229

[45] Al-Refaie WB, Parsons HM, Henderson WG, et al. Major cancer surgery in the elderly: Results from the american college of surgeons national surgical quality improvement program. Ann Surg. 2010;251(2):311–318. doi: 10.1097/SLA.0b013e3181b6b04c 19838107

[46] Weinberg L, Li MHG, Churilov L, et al. Associations of Fluid Amount, Type, and Balance and Acute Kidney Injury in Patients Undergoing Major Surgery. Anaesth Intensive Care. 2018;46(1):79–87. doi: 10.1177/0310057X1804600112 29361260

[47] Privratsky JR, Krishnamoorthy V, Raghunathan K, et al. Postoperative Acute Kidney Injury Is Associated With Progression of Chronic Kidney Disease Independent of Severity. Anesth Analg. 2022;134(1):49. doi: 10.1213/ANE.0000000000005702 34908546

[48] Strijack B, Mojica J, Sood M, et al. Outcomes of chronic dialysis patients admitted to the intensive care unit. J Am Soc Nephrol JASN. 2009;20(11):2441–2447. doi: 10.1681/ASN.2009040366 19729437 PMC2799175

[49] Pattakos G, Johnston DR, Houghtaling PL, Nowicki ER, Blackstone EH. Preoperative prediction of non-home discharge: A Strategy to reduce resource use after cardiac surgery. J Am Coll Surg. 2012;214(2):140–147. doi: 10.1016/j.jamcollsurg.2011.11.003 22265219

[50] Hauck K, Zhao X. How dangerous is a day in hospital?: A Model of adverse events and length of stay for medical inpatients. Med Care. 2011;49(12):1068. doi: 10.1097/MLR.0b013e31822efb09 21945976

