# Supplementary material for: Association of chronic kidney disease with acute clinical outcomes and hospitalization costs of cancer resection
Source: PLoS One. 2025 Jan 24;20(1):e0317085. doi: 10.1371/journal.pone.0317085 (PMC11760676; doi:10.1371/journal.pone.0317085)
Supplement: S1 Table — Outcomes reported as proportion (%). *IQR, interquartile range; USD, United States dollar; CKD, Chronic Kidney Disease; ESRD, End-Stage Renal Disease. (DOCX) [file pone.0317085.s001.docx]

**SUPPLEMENTARY APPENDIX**

**Association of Chronic Kidney Disease with Acute Clinical Outcomes and Hospitalization Costs of Cancer Resection**

Sara Sakowitz MS MPH^1^, Syed Shahyan Bakhtiyar MD MBE^1,2^, Saad Mallick MD^1,3^

Amulya Vadlakonda BS^1^, Ifigenia Oxyzolou^1^, Konmal Ali^1^, Nikhil Chervu MD^1,3^, Peyman Benharash MD MS^1,3^

^1^ Cardiovascular Outcomes Research Laboratories (CORELAB), Department of Surgery, University of California, Los Angeles, Los Angeles, CA

^2^ Department of Surgery, University of Colorado, Aurora, CO

^3^ Department of Surgery, University of California, Los Angeles, Los Angeles, CA

**Corresponding Authors:**

Peyman Benharash MD MS [pbenharash@mednet.ucla.edu](mailto:pbenharash@mednet.ucla.edu)

Sara Sakowitz MS MPH [ssakowitz@mednet.ucla.edu](mailto:ssakowitz@mednet.ucla.edu)

**Contents**

[**SUPPLEMENTAL TABLE S1: Unadjusted Patient Outcomes Stratified by Degree of CKD** 3](#_Toc185866316)

# **SUPPLEMENTAL TABLE S1: Unadjusted Patient Outcomes Stratified by Degree of CKD**

Outcomes reported as proportion (%).

**IQR*, interquartile range; *USD,* United States dollar; *CKD*, Chronic Kidney Disease; *ESRD,* End-Stage Renal Disease

|  | ***Non-CKD*** | ***CKD 1-3*** | ***CKD 4-5*** | ***ESRD*** | ***P-value*** |
| --- | --- | --- | --- | --- | --- |
| **Clinical outcomes** |  |  |  |  |  |
| In-hospital mortality | 1.1 | 2.1 | 3.3 | 5.4 | <0.001 |
| Acute kidney injury | 5.7 | 25.3 | 41.7 | - | - |
| Cardiac complications | 1.0 | 2.1 | 3.1 | 3.1 | <0.001 |
| Infectious complications | 3.0 | 4.0 | 7.3 | 10.1 | <0.001 |
| Respiratory complications | 6.8 | 9.3 | 12.3 | 14.5 | <0.001 |
| Blood transfusion | 5.7 | 10.4 | 14.7 | 13.6 | <0.001 |
| Thrombotic complication | 0.7 | 0.6 | 1.0 | 1.0 | 0.41 |
| Stroke complications | 0.2 | 0.4 | 0.4 | 0.4 | 0.001 |
| Non-home discharge | 7.9 | 19.0 | 25.2 | 26.8 | <0.001 |
| **Resource utilization** |  |  |  |  |  |
| Length of stay (days) [IQR] | 5 [3-7] | 6 [4-8] | 6 [4-10] | 6 [4-11] | <0.001 |
| Cost (USD $1,000) [IQR] | 21.6  [15.2-32.3] | 23.9  [16.7-35.9] | 25.2  [17.7-37.2] | 26.9  [18.5-46.0] | <0.001 |
